# Beginning of the End (BOTE) Sign in the Setting of Recurrent Molluscum Contagiosum in a Child

**DOI:** 10.7759/cureus.89489

**Published:** 2025-08-06

**Authors:** Remi T Parker, Julissa A Jimenez, Thy Huynh, Robert T Brodell

**Affiliations:** 1 Pediatrics, University of Tennessee Health Science Center, Memphis, USA; 2 Dermatology, University of Mississippi Medical Center, Jackson, USA; 3 Pathology, Dermatology, University of Mississippi Medical Center, Jackson, USA

**Keywords:** bote sign, cutaneous inflammation, expectant management, immunologic skin response, molluscum contagiosum, non-invasive dermatologic treatment, pediatric dermatology, self-limited dermatoses, viral dermatoses

## Abstract

Molluscum contagiosum (MC) is a common cutaneous viral infection predominantly affecting children. In this report, we present the case of a five-year-old male with recurrent MC who developed the beginning of the end (BOTE) sign, reflecting an inflammatory response that correlates with imminent lesion resolution. The patient’s lesions were monitored without further intervention following the appearance of the BOTE sign, and complete resolution was documented in roughly two months. Serial photographs taken by the patient’s parent provided visual documentation of the natural disease progression and healing of the lesions. Our case underscores the importance of distinguishing the BOTE sign from secondary bacterial infection and highlights its value in predicting spontaneous resolution, allowing clinicians to confidently opt for conservative management.

## Introduction

Molluscum contagiosum (MC) is one of the most prevalent cutaneous infections worldwide. It is a DNA poxvirus that predominantly appears in children, young adults, and immunocompromised patients [1]. The infection is frequently transmitted through skin-to-skin contact, fomites, or sexual contact [1-4]. Patients with a history of atopic dermatitis seemingly also have an increased susceptibility to MC [2].

On skin examination, the rash associated with MC commonly appears as defined, dome-shaped, skin-colored papules that are typically less than 5 mm in diameter [4, 5]. The infection may present as individual lesions or grouped lesions in a linear or clustered pattern. The lesions can also develop pruritus, scaling, and tenderness to palpation [5]. The incubation period from exposure to first signs of infection is two to eight weeks, with the total duration of the infection until resolution of symptoms ranging roughly from two months up to three years [1, 3]. In this report, a child with recurrent MC is presented, highlighting the beginning of the end (BOTE) sign, an inflammatory indicator of impending lesion regression, documented with lesional photography.

## Case presentation

A five-year-old male in the southeast United States with a past medical history of torticollis presented to the dermatology clinic with a two-month history of two dozen nontender umbilicated papules 0.5 to 1.5 mm in diameter on the lower extremity, penile shaft, and buttocks. MC first appeared in our patient two years earlier with lesions of the left upper extremity, abdomen, and right lower extremity. His sister had similar lesions at the same time. The patient and his parents denied a history of trauma or abuse. Parents denied any known allergies or significant family medical history, and there was no history of an immunodeficiency syndrome. Our patient was up to date on all vaccinations. After shared decision-making with our patient’s parents, all visible lesions were curetted following application of eutectic mixture of local anesthetics (EMLA), which is composed of lidocaine 2.5% and prilocaine 2.5%. The lesions healed rapidly with Vaseline ointment applied to wounds twice daily.

**Figure 1:**
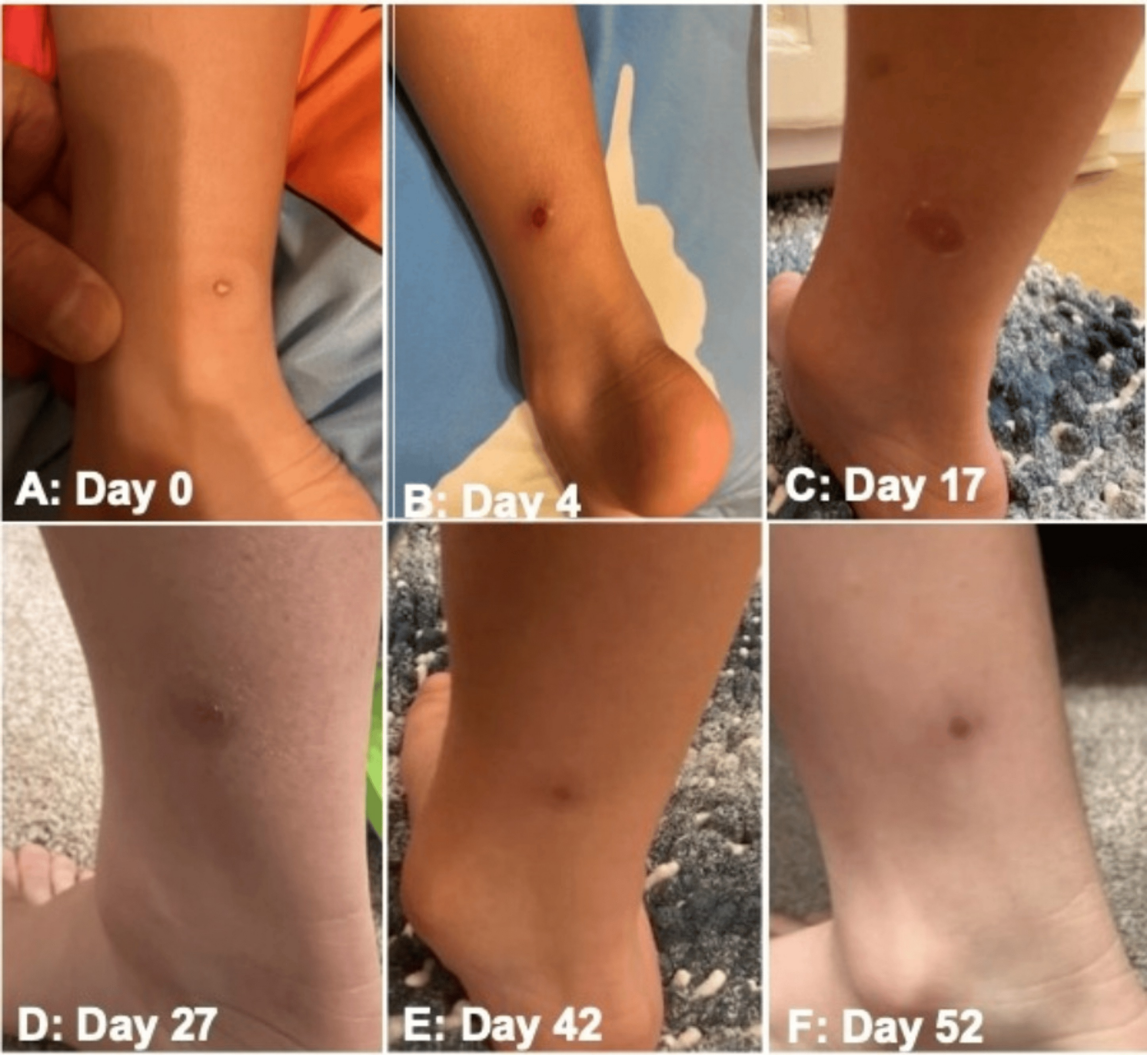
Time lapse of lesion on posterior aspect of right leg from the inflammatory to post-inflammatory phases A) Day 0: Day 0: 1–1.5 mm slightly erythematous papule with a central punctum, suggestive of an early inflammatory papule consistent with the beginning of the end (BOTE) sign. No visible induration or surrounding erythema. Surrounding hypopigmentation was from a spot band-aid that prevented tanning. (B) Day 4: The lesion evolved into a well-demarcated, centrally crusted papule with surrounding erythema and mild induration, indicating progression of the localized inflammatory response. (C) Day 17: Flattened violaceous plaque with subtle central clearing and residual induration, consistent with evolving resolution of the inflammatory process and early regression. (D) Day 27: Faint, violaceous macule with minimal elevation and no active erythema, demonstrating marked improvement. Induration had resolved without signs of superinfection. (E) Day 42: The lesion continued to flatten and fade, now presenting as a lightly pigmented macule with subtle post-inflammatory hyperpigmentation. No induration or erythema. (F) Day 52: Complete clinical resolution was noted with mild residual post-inflammatory erythema and no palpable abnormality.

**Figure 2:**
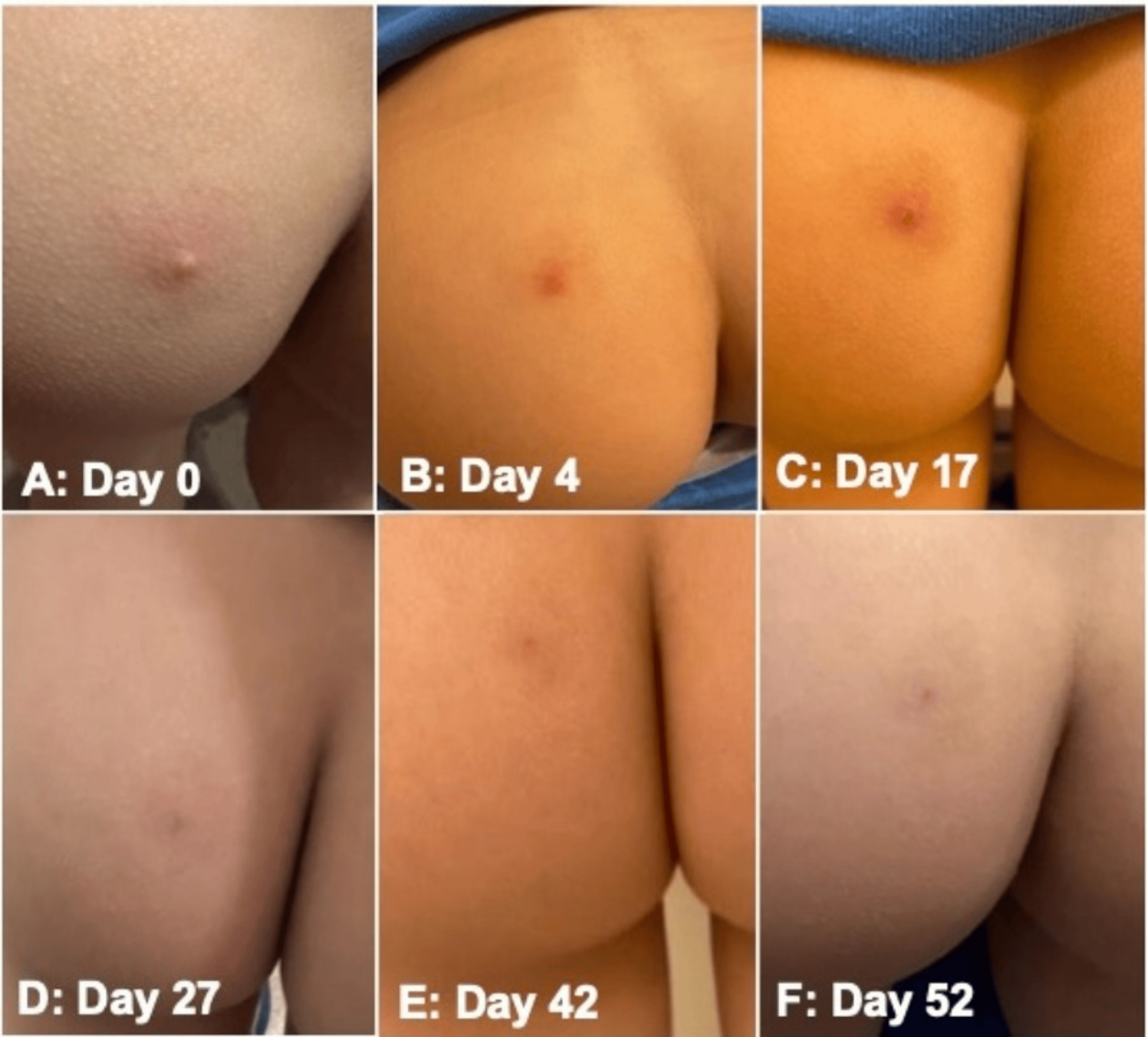
Time lapse of lesion on posterior left buttock from the inflammatory to post-inflammatory phases (A) Day 0: Firm, skin-colored papule with a central pinpoint depression and mild surrounding flare of erythema, consistent with an early inflammatory lesion. (B) Day 4: Flattened erythematous papule with surrounding erythema and a smoother surface, suggesting early resolution of the acute inflammatory phase. No visible pustule or scale crust was present. (C) Day 17: Continued flattening of papule, with central, pinpoint crust and dusky erythema consistent with progressive involution. (D) Day 27: Faintly erythematous macule with only slight central elevation consistent with post-inflammatory change. (E) Day 42: Subtle pinkish macule, demonstrating continued regression of the lesion. (F) Day 52: Complete resolution of the lesion with only faint post-inflammatory erythema. No induration, scale, or pigment alteration, indicating restoration of normal skin integrity.

At the next visit, two years later, physical examination revealed inflamed and shiny, skin-colored 1-1.5 mm umbilicated papules present on the right lower extremity, penile shaft, and the left buttock with inflammation of several lesions consistent with the BOTE sign that appeared within the previous two weeks (Figures 1-2). No further treatment was recommended, considering the self-limited nature of this infectious process in the setting of the BOTE sign. Ten days after the lesions became inflamed, the patient’s father began taking serial daily photos of the inflamed MC lesions on the leg and the left buttock to assess the evolution of the lesions over time (Figures 1-2; complete panel of images documented in Appendix A, B). Complete resolution of the lesions occurred roughly 63 days after the BOTE sign appeared.

## Discussion

MC lesions appear as well-defined, dome-shaped, skin-colored papules that are typically less than 5 mm in diameter [4,5]. The infection may present as a single lesion or as a cluster. While generally asymptomatic, patients may experience tenderness or pruritus at the site [3]. Pseudo-koebnerization can also produce a linear pattern. The incubation period from exposure to first signs of infection is two to eight weeks, with the total duration of the infection until resolution of symptoms ranging roughly from two months up to three years [1,3]. As stated above, once the natural immune process induces the beginning of spontaneous resolution, the BOTE sign emerges. This sign may present with erythema, edema, induration, scale, and crusting of lesions [5]. The molluscum papules may develop a punctum/central umbilication during the process of regression [6]. The BOTE sign may initially be associated with tenderness or pruritus, but these symptoms usually resolve as the lesion involutes.

Though crusting or pustulation of molluscum lesions may appear when spontaneous resolution is imminent, the inflammation and edema of the BOTE sign should not be mistaken for bacterial superinfection, which most commonly is associated with purulent drainage, honey-colored crusting, or follicular pustules (bacterial folliculitis) [3,7]. Worsening bacterial superinfection may show expanding erythema, warmth, purulent exudate, or systemic manifestations such as fever and chills [7]. Wound cultures can help avoid unnecessary antibiotics in cases where impetigo, folliculitis, or cellulitis are suspected.

The BOTE sign can be seen in verruca plana infections due to human papillomavirus as well, which could clinically be confused with MC [5]. The differential diagnosis also includes disseminated *Cryptococcus neoformans* in immunocompromised patients [2,5]. If the diagnosis is unclear from the history and examination findings, histologic examination of curetted lesions can be used to detect the Henderson-Patterson bodies characteristic of MC, which are enlarged keratinocytes with cytoplasmic inclusions residing in hyperplastic epithelial tissue [1,6,8].

The majority of MC infections resolve spontaneously, and a “wait and see” approach is most common, particularly among pediatricians [9]. However, more recently, literature has emerged that favors active treatment of the infection as opposed to expectant management to reduce discomfort, prevent spread of infection to other individuals, and decrease social stigma associated with the lesions (Table 1) [2,4,5,9]. Patients with recurring lesions or persistent primary infection may undergo procedural treatment, such as curettage, cryotherapy, or incision and drainage. The Food and Drug Administration recently approved an immunomodulator, SB206 (Berdazimer 10.3% gel), and a blistering agent (cantharidin) for the treatment of MC [10,11]. These less invasive options may be more readily adopted in the United States to reduce potential scarring from the procedural therapies [1,10]. A recent study suggested that the use of topical immunomodulators, specifically SB206 Berdazimer gel, may promote quicker resolution in patients with the BOTE sign [12]. 

**Table 1:** Considerations for pursuing active treatment in molluscum contagiosum (MC) infections in contrast to the conservative approach Table credits: Remi Parker (author); Sources: [2, 4 -5, 10]

Treatment approach	Advantages	Disadvantages
Active treatment	Quicker resolution of lesions	Increased risk of scarring and keloid formation
	Prevents viral infection of other people	An added financial expense
	Provides symptomatic relief	Potential exacerbation of the local inflammatory response
	May improve the appearance of lesions, resulting in improved confidence	Increased risk of bacterial superinfection due to treatment-related loss of skin barrier integrity
Conservative management	Cost effective	It may take up to three years to resolve spontaneously
	Avoids unnecessary overtreatment	Increased risk of viral spread to other people
	Avoids aggravating the inflammatory process	Increased social stigmatization is associated with persistent lesions

Despite previous successful, albeit transient, treatment by curettage, the presence of the BOTE sign led to expectant monitoring of the lesions, which resolved over the course of two months in our patient. In 2013, Butala et al. suggested that 3.6 months was the average time from the onset of inflammation to complete resolution of lesions, with a range of three weeks to five months [3]. In 2021, Maeda-Chubachi proposed that it takes less than six months for complete resolution of symptoms; however, their study was performed in the setting of SB206 gel administration [12]. Their study also did not specify whether the hypothesized duration is measured from the onset of MC lesions or the appearance of the BOTE sign. With our report, we update the literature and support both of the previous studies’ claims with documentation of our patient’s resolution of symptoms roughly two months after the BOTE sign developed. We believe that pursuing expectant management in our patient’s recurrent MC lesions in the presence of the BOTE sign enabled us to avoid iatrogenic irritation of the lesions that may have delayed healing and to ultimately respect the natural course of the viral process.

## Conclusions

This case highlights the clinical significance of the BOTE sign as an indicator of spontaneous resolution in MC infections, particularly in the pediatric setting. The documented progression and eventual clearance of lesions in this five-year-old patient supports the utility of a conservative, observation-based management approach when the BOTE sign manifests. Our case reinforces prior literature suggesting that the inflammatory response associated with the BOTE sign should not be mistaken for secondary infection, thereby avoiding unnecessary interventions. Ultimately, we propose that prompt recognition of the BOTE sign can guide clinicians in making informed decisions between active treatment and expectant management, potentially minimizing patient discomfort and overtreatment.

## Figures and Tables

**Figure 3:**
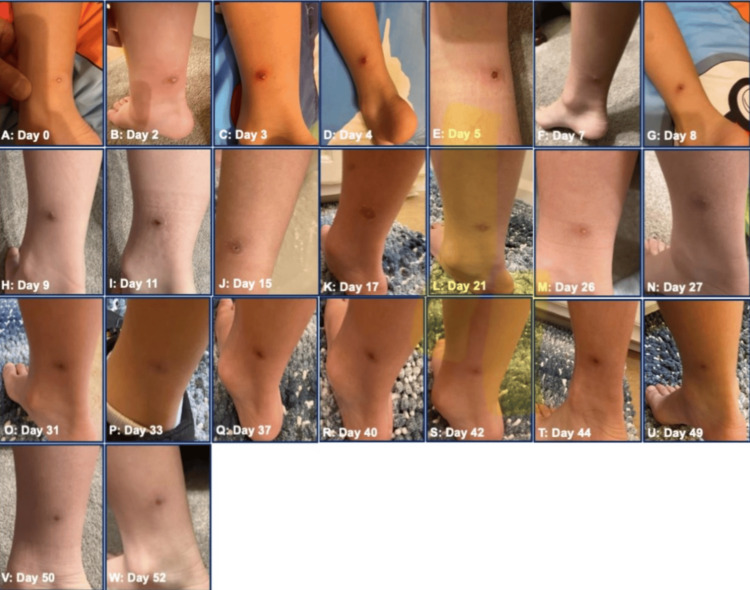
Time lapse of lesion on posterior aspect of right leg from the inflammatory to post inflammatory phases. Some transition points include: (A) Day 0: 1–1.5 mm erythematous papule with a central punctum, suggestive of an early inflammatory papule consistent with the BOTE sign. No visible induration or surrounding erythema. (B) Day 4: The lesion has evolved into a well-demarcated, centrally ulcerated papule with surrounding erythema and mild induration, indicating progression to a localized inflammatory response. (C) Day 17: Flattened violaceous plaque with subtle central clearing and residual induration, consistent with evolving resolution of the inflammatory process and early regression. (D) Day 27: Faint, violaceous macule with minimal elevation and no active erythema, demonstrating marked improvement. Induration has resolved without signs of superinfection. (E) Day 42: The lesion continues to flatten and fade, now presenting as a lightly pigmented macule with subtle post-inflammatory hyperpigmentation. No induration or erythema. (F) Day 52: Complete clinical resolution with residual post-inflammatory hyperpigmentation and no palpable abnormality. Skin texture and pigmentation continue to normalize. BOTE: beginning of the end

**Figure 4:**
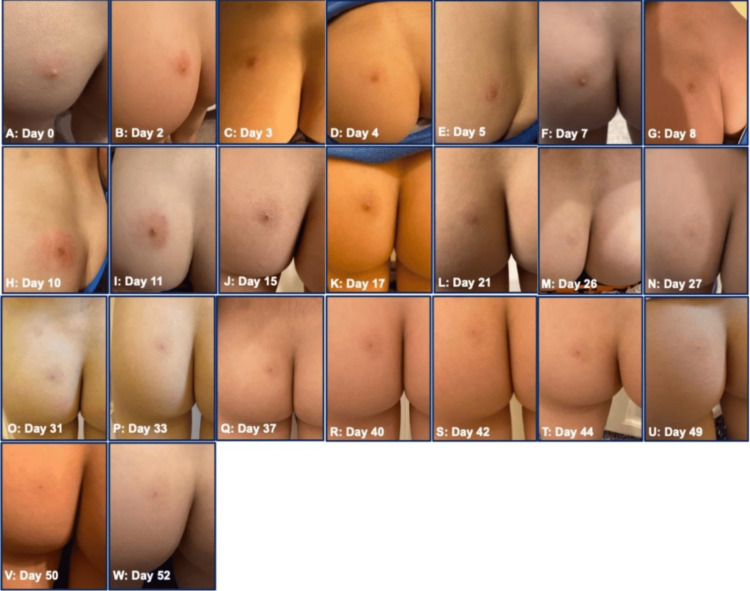
Time lapse of lesion on posterior left buttock from the inflammatory to post-inflammatory phases. Time lapse of lesion on posterior left buttock from the inflammatory to post-inflammatory phases. Some transition points include: (A) Day 0: Firm, erythematous papule with a central pinpoint elevation, consistent with an early inflammatory lesion. No surrounding erythema or drainage is appreciable. (B) Day 4: Flattened erythematous papule with slight surrounding erythema and a smoother surface, suggesting early resolution of the acute inflammatory phase. No visible pustule or crust formation. (C) Day 17: Continued flattening of papule, with central dusky erythema and diminished induration indicating progressive involution. (D) Day 27: Faintly erythematous macule without palpable elevation or induration, consistent with post-inflammatory change. (E) Day 42: Subtle pinkish macule, demonstrating continued regression of the lesion. No signs of active inflammation or residual thickening. (F) Day 52: Complete resolution of the lesion with only faint post-inflammatory erythema. No induration, scale, or pigment alteration, indicating impending restoration of normal skin integrity.

## Appendices

Appendix A 

Appendix B 
